# Fostering emotional, social, physical and educational wellbeing in rural India: the methods of a multi-arm randomized controlled trial of Girls First

**DOI:** 10.1186/s13063-015-1008-3

**Published:** 2015-10-26

**Authors:** Katherine Sachs Leventhal, Lisa M. DeMaria, Jane Gillham, Gracy Andrew, John W Peabody, Steve Leventhal

**Affiliations:** CorStone, 250 Camino Alto, Suite 100A, Mill Valley, CA USA; QURE Healthcare, 1000 Fourth St., Suite 300, San Rafael, CA USA; Department of Psychology, Swarthmore College, 500 College Avenue, Swarthmore, PA USA; CorStone India Foundation, c/o Ajay K. Sud & Associates, B-4 Greater Kailash Enclave, Part-II, New Delhi, 110048 India; QURE Healthcare, QURE Healthcare, 1000 Fourth St., Suite 300, San Rafael, CA USA

**Keywords:** Resilience, Adolescents, Physical wellbeing, Emotional wellbeing, Social wellbeing, Educational wellbeing, Intervention, India, Girls, Methodology, Randomized controlled trial

## Abstract

**Background:**

There are 600 million girls in low and middle income countries (LMICs), many of whom are at great risk for poor health and education. There is thus great need for programs that can effectively improve wellbeing for these girls. Although many interventions have been developed to address these issues, most focus on health and education without integrating attention to social and emotional factors. This omission is unfortunate, as nascent evidence indicates that these factors are closely related to health and education.

This paper describes the methods of a 4-arm randomized controlled trial among 3,560 adolescent girls in rural Bihar, India that tested whether adding an intervention targeting social-emotional issues (based on a “resilience framework”) to an adolescent health intervention would improve emotional, social, physical, and educational wellbeing to a greater extent than its components and a control group. Study arms were: (1) Girls First, a combination of the Girls First Resilience Curriculum (RC) and the Girls First Health Curriculum (HC); (2) Girls First Resilience Curriculum (RC) alone; (3) Girls First Health Curriculum (HC) alone; and (4) a school-as-usual control group (SC).

**Methods:**

Seventy-six schools were randomized (19 per condition) and 74 local women with a tenth grade education were trained and monitored to facilitate the program. Quantitative data were collected from 3,560 girls over 4 assessment points with very low rates of participant attrition. Qualitative assessments were conducted with a subset of 99 girls and 27 facilitators.

**Results and conclusions:**

In this article, we discuss guiding principles that facilitated trial implementation, including integrating diverse local and non-local sources of knowledge, focusing on flexibility of planning and implementation, prioritizing systematic measurement selection, and striking a balance between scientific rigor and real-world feasibility.

**Trial registration:**

Clinicaltrials.gov NCT02429661. Registered 24 April 2015.

**Electronic supplementary material:**

The online version of this article (doi:10.1186/s13063-015-1008-3) contains supplementary material, which is available to authorized users.

## Background

There are 600 million girls in low and middle income countries (LMICs) [1], many of whom are at great risk for poor physical and mental health and education outcomes. Globally, girls make up two thirds of child-trafficking victims [2], are more likely to be out of school than boys [3], and are more likely than boys to be forced into marriages before age 18, leading them to high risk for domestic abuse and the dangers of teenage pregnancy [4]. Girls and women are also more likely than boys and men worldwide to face psychological problems such as depression and anxiety [5–9].

Many have recognized these concerns, creating programs specifically to empower adolescent girls in LMICs. Currently, many programs focus on imparting physical health-related knowledge and behaviors, encouraging girls to stay in school, or teaching girls vocational skills. While various pedagogies have been used, only nascent evidence supports these interventions, and many gaps remain in our knowledge [10–12].

There is thus great need for innovative interventions that address these issues, and an equally substantial need for rigorous evaluations to establish an evidence base supporting health and education improvements among adolescent girls in LMICs. However, developing such interventions and conducting rigorous evaluations in LMICs can be highly challenging. For instance, human resources required to facilitate programs are often scarce or unavailable locally; infrastructure may be so poor and distances so vast that scheduling, monitoring, and support of interventions become great challenges; measurements used elsewhere to quantify intervention impact may be culturally or linguistically inappropriate; major variations in cultural and linguistic environments can make broadly implementing interventions difficult; and cultural attitudes about girls’ responsibilities, which restricts their mobility, may make them difficult to access.

In this paper, we describe the methodology we used in designing and testing a promising intervention to improve girls’ emotional, social, physical and educational wellbeing in rural India, including the major challenges and lessons learned, which we hope will help others to feel empowered and supported to conduct similar trials in similarly challenging settings.

### Rationale of the innovation

Research in higher-income countries (HICs) confirms that “resilience,” or the ability to bounce back and grow from challenges or crises, is consistently related to positive emotional, social, physical, and educational wellbeing for at-risk youth [13–15]. Additionally, studies suggest that resilience can be built by developing assets such as self-efficacy, self-awareness, coping and decision-making skills, communication skills, and strong bonds with peers and family [16–18]. Many resilience-based and related interventions to improve such social and emotional assets have been conducted in HICs in schools because they provide a ready and structured means of access to adolescents [19, 20]. Adopting a “resilience-based framework” that promotes these assets in school-based programs for LMIC girls could, therefore, broadly impact wellbeing. Despite the potential for positive outcomes, few rigorously-evaluated programs in LMICs have focused on improving resilience [21–23].

The limited evidence of such LMIC programs that does exist, however, suggests positive effects [23, 24]. For instance, a recent review of school-based programs to promote social and emotional skills in LMICs noted that results were generally positive for emotional and social outcomes, and the few programs that measured physical and educational outcomes also noted positive effects on school adjustment, fitness, attitudes about reproductive and sexual health, and substance use [23].

As emotional and social factors have been shown to be integrally related to the ability to overcome physical-related and education-related risk factors [13, 14], it is critical to investigate whether adding a resilience component to interventions that target physical health and academic success could improve outcomes in these areas for populations in LMICs.

### Present study

In response, since 2009, CorStone, a US-based non-profit organization, has developed and piloted a resilience-based program, called Girls First, to improve emotional, social, physical and educational wellbeing for adolescent girls in India. The full Girls First intervention includes two components: the Girls First Resilience Curriculum (RC) that targets social and emotional assets and wellbeing, and the Girls First Health Curriculum (HC) that directly targets adolescent physical health and wellbeing. This paper presents details on the intervention and the methodology of the current study, which is a multi-arm randomized controlled trial (RCT). Our goal was to investigate whether the full Girls First program had a greater effect than its components alone on emotional, social, physical, and educational wellbeing (for trial results see for instance [25, 26]).

Before this study, two pilots of the RC suggested that it was feasible, acceptable, and beneficial to adolescent girls in India. The first study, an open, uncontrolled trial in Delhi, included approximately 100 girls aged 12–18. The proportion of girls with normal mental health scores on the Strengths and Difficulties Questionnaire (SDQ) [27] increased significantly from 52.6 % (pre-intervention) to 63.9 % (post-intervention). The second pilot compared RC groups with matched controls among approximately 900 girls in 4 government schools in urban slums in Surat, India. In this trial, the percentage of girls with “normal” SDQ scores significantly increased among girls receiving RC from pre-intervention to post-intervention. The control group did not change significantly.

Physical and educational wellbeing, and the program’s likely synergistic relationship with an adolescent physical health intervention, were not assessed in either of these trials. Thus, though RC appeared to have positive effects, an important next step was to investigate the effects of RC in combination with an adolescent physical health curriculum (HC) on girls’ emotional, social, physical, and educational wellbeing in an adequately-powered, controlled experimental study to begin to determine causality and magnitude of change in outcomes.

The main objective of the current study is to compare the effects of the combined curriculum (Girls First or RC + HC) to its components (RC only and HC only) and a control group (SC) in these four outcome domains. The study hypotheses are primarily that RC + HC would have a greater effect in these domains than the other conditions. Secondary hypotheses are that RC would have greater effects on emotional and social wellbeing than HC or SC, and HC would have greater effects on physical wellbeing than RC or SC. Because both RC and HC target factors related to educational outcomes, RC and HC are expected to be more beneficial than SC for these outcomes.

## Methods

*Girls First – Bihar* was a four-arm RCT of a resilience-based program to empower adolescent girls in rural India with knowledge, skills and support to improve their emotional, social, physical, and educational wellbeing. The project was conducted by CorStone, a US-based organization.

## Trial design

The trial combined two curricula: an emotional resilience-building curriculum (RC) with an adolescent health curriculum (HC). These curricula were tested as follows: the (a) combined program (RC + HC or “Girls First”), was compared to (b) the Girls First Health Curriculum alone (HC), (c) the Girls First Resilience Curriculum alone (RC), and (d) a school-as-usual control condition (SC). Outcomes spanned four domains of wellbeing: emotional, social, physical, and educational. Assessments were mixed-methods, employing quantitative and qualitative measures. The full study was approved and overseen by Chesapeake Institutional Review Board (Columbia, MD, USA) and Sangath Institutional Review Board (Goa, India).

### Quantitative component sampling method

The study sample was adolescent girls aged 9–18 in VII–VIII standards (Stds.; equivalent to US seventh and eighth grades) in rural Bihar, India.^1^ According to Oxford’s Multidimensional Poverty Index, 79 % of Bihar’s population lives in poverty [28]. In Bihar, girls are greatly at-risk; for instance, 95 % of women have less than 12 years of education, 64 % of girls are married before age 18, and 45 % of women aged 15–49 have experienced physical or sexual violence [29].

Two local partner organizations were selected to train to implement Girls First, based on their strong community presences: GENVP (Gramin Evam Nagar Vikas Parishad) and IDF (Integrated Development Foundation), in Patna, Bihar. GENVP and IDF recommended three rural blocks of Patna District for the study based on their previous experience: Phulwarisharif, Maner, and Bihta (in Bihar, a block is a sub-division of a district, composed of multiple villages).

### Sampling process

GENVP and IDF compiled a list of 86 schools which comprised the initial sampling frame. Inclusion criteria for the initial sampling frame were feasibility and accessibility, defined by GENVP’s and IDF’s opinion of whether they, as organizations with a strong and broad community presence, would be able to access and work with these schools to conduct and monitor the study interventions.

From the sampling frame of 86 schools, the only study inclusion criterion was that combined girl enrollment in VII–VIII Stds. be between 20–150 girls.^2^ Sixty-nine schools met this criterion. One of these 69 schools was then excluded by request of its principal. Thus, 68 schools were initially selected for the study and conducted their first assessments (Time 1) starting in July 2013. Once the study began, girls’ actual enrollment was found to be much lower than official school records reported; therefore, 11 more schools were selected in which GENVP and IDF deemed it possible to work. All of these schools had between 20–150 girl students, but it was only possible to retain 8 based on funding constraints. Thus, eight of these eleven were randomly selected for study inclusion. These new schools completed the study procedures at a lag of approximately 6 months.

### Consent process and assessments

All schools in the study completed the consent process and all 4 time points of the assessments (Time 1 (T1), Time 2 (T2), Time 3 (T3), and Time 4 (T4)), with the exception of the 8 schools added later to the trial. These 8 schools only completed T1, T2, and T3, and did not complete T4 due to time constraints.

**Fig. 1:**
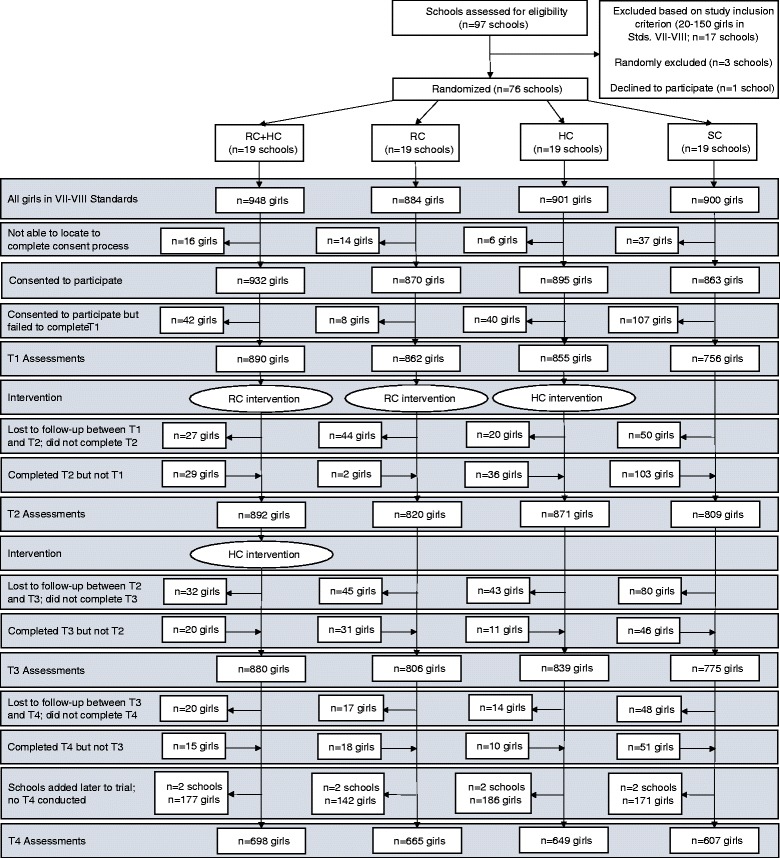
Consolidated Standards of Reporting Trials (CONSORT) diagram. Legend: HC, Girls First Health Curriculum; RC, = Girls First Resilience Curriculum; SC, school-as-usual control; T1, Time 1; T2, Time 2; T3, Time 3; T4, Time 4

Figure 1 provides the study Consolidated Standards of Reporting Trials (CONSORT) diagram. Informed consent was obtained from all participants. All 3633 girls in Stds. VII–VIII at the 76 schools were invited to participate in the study. Three thousand five hundred and sixty girls completed the consent process (including both parental consent and child assent). Three thousand three hundred and sixty-three completed T1 (93 % of girls who consented). Three thousand three hundred and ninety-two completed T2 (93 % of girls who consented), 3300 completed T3 (91 % of girls who consented), and 2619 completed T4 (91 % of girls who consented in the 68 schools where T4 was conducted).

### Sample size calculations

Three primary outcomes areas were considered for this study: health (emotional and physical), education, and social. Sample size calculations are based on the sample frame and sample size using Generalized Estimating Equations. We used findings from previous studies in India in which we were able to detect effects of at least 7–8 % on similar indicators used in this study [30]. Assuming that there is no change from baseline in the control group during the post-intervention period, previous studies show a SD of 12 % within group variance and a 5 % variance between groups. With a 2-sided significance level of 0.05 and a desired power of 80 %, it was found that 19 schools per arm with 40 students per school would be more than adequate.

### Quantitative component randomization

Blocked randomization was conducted, stratifying schools by school block (geographical location) and size of school. The pool of schools within each strata was then selected (e.g., schools in Phulwarisharif block that were above average in size for that school block) and randomized in blocks of eight such that within each block of eight, two schools were assigned to each condition. After randomization, study arms did not differ significantly on stratification variables.

### Interventions

The Girls First intervention is based on evidence from fields including: (1) positive psychology [31], (2) emotional competence [32], (3) restorative practices [33–36], (4) global adolescent health [37], and (5) peer support [38–40].

Girls First is made up of 2 interventions: RC, which is comprised of 23 hour-long weekly facilitated peer support group sessions, and HC, which is comprised of 21 hour-long weekly facilitated peer support group sessions. The topics covered are detailed in Table 1. Additional details about the curricula can be found in Appendix A (see Additional file 1).

**Table 1:** Topics covered during intervention sessions

Resilience Curriculum (RC) sessions		Health Curriculum (HC) sessions	
Session 1	Introduction and assessments	Session 1	Introduction and assessments
Session 2	Setting group guidelines	Session 2	The health system
Session 3	Listening skills	Session 3	Nutrition and anemia (I)
Session 4	Character strengths (I)	Session 4	Nutrition and anemia (II)
Session 5	Character strengths (II)	Session 5	Water, sanitation and health
Session 6	Life stories and goals	Session 6	Key health issues
Session 7	Planning to reach our goals	Session 7	Diarrhea and diarrhea management
Session 8	Identifying emotions	Session 8	Review
Session 9	Emotional awareness	Session 9	Gender constructs (I)
Session 10	Managing strong emotions (I)	Session 10	Gender constructs (II)
Session 11	Benefit finding	Session 11	Know your body
Session 12	Managing strong emotions (II)	Session 12	The reproductive system
Session 13	Assertive communication	Session 13	Menstruation and hygiene
Session 14	Restorative practices for conflict resolution	Session 14	My relationships
Session 15	Group problem solving	Session 15	Intimate relationships
Session 16	Identifying and opposing violence	Session 16	Physical intimacy
Session 17	Forgiveness and apologies	Session 17	Gender-based violence
Session 18	Self-esteem and character strengths	Session 18	Understanding and promoting rights
Session 19	Problem solving with a focus on friendships	Session 19	Substance use and abuse
Session 20	Peace project (I)	Session 20	Review and celebrate
Session 21	Peace project (II)	Session 21	Assessments
Session 22	Review and celebrate		
Session 23	Assessments and gratitude		

Schools assigned to Girls First (RC + HC) received RC then HC. Schools assigned to other intervention arms (RC or HC) completed only one component of the curriculum. In order to keep curriculum components constant across study conditions, girls in the RC + HC condition therefore attended more sessions over a longer time period than girls in RC or HC. Schools assigned to SC did not receive either component.

Group schedules were decided with school administrators on a case-by-case basis. As all 57 schools in the 3 intervention arms opted to conduct the groups during the school day, SC girls received 1 additional hour of school each week compared with girls in the intervention conditions.

### Training and implementation

All program components were provided in in-school facilitated peer-support groups comprised of approximately 12–15 girls per group, combining didactic learning with peer-led discussion and problem-solving. On average, girls attended 74 % of sessions at their schools (SD = 24 %).

Seventy-four local women with at least a standard X education (Std. X; equivalent to a tenth grade education in the US school system) served as facilitators to lead groups. Facilitators were recruited through postings in local public spaces and job postings throughout IDF’s and GENVP’s networks. Facilitators were required to be women aged 18 or older, have at least a Std. X education, and live in local communities. Seventy-three facilitators were initially recruited, and one additional facilitator was added part of the way through the study. Five facilitators left the project either for personal or performance reasons before the study’s completion. Their groups were taken over by other trained facilitators.

CorStone trained four Master Trainers, who in turn provided training on facilitation and program content for facilitators. Master Trainers were recruited through IDF’s, GENVP’s and CorStone’s professional networks, and were required to be women with at least a master’s level education who could live in Patna, Bihar for the duration of the study. Both Master Trainers and facilitators were provided with a stipend.

The RC training for facilitators consisted of a 5-day initial training and a 3-day follow-up training mid-way through the program. The HC training consisted of a 3-day initial training and a 3-day follow-up mid-way through the program. These trainings were developed in consultation with local staff with the goal of preparing facilitators to have adequate mastery of the curricula. Given the novelty of the concepts, local staff recommended that 2 additional days would be required in RC training for facilitators to reach the same level of mastery as those in HC training. Additionally, Master Trainers provided facilitators with support, review and training every 2 weeks (through group meetings) throughout the program to maintain quality and fidelity.

Facilitators co-facilitated weekly groups in pairs. The 25 facilitators in RC + HC were trained in both curricula. The 26 facilitators in RC generally were trained only in RC, though 5 RC facilitators were also trained in HC as they also conducted some HC groups because of scheduling, travel, and other logistical issues. The 23 facilitators in HC were generally trained only in HC, with the exception of 2 facilitators who were trained in RC for the same reasons.

### Intervention monitoring, standardization and adherence

Facilitators were provided with manuals during their trainings that they then used during sessions as a means of intervention standardization. Manuals included the curriculum and steps to be followed during each session. Master Trainers closely monitored implementation using observation forms and protocols. Monitoring protocols included observations of adherence to the curricula in the manuals as well as facilitator ability to adequately deliver curricula and manage participants. Master Trainers observed facilitators once every month throughout the intervention. Facilitators who were found to need additional support during monitoring visits received additional training during refresher trainings.

### Assessments

Girl participants completed assessments at 4 time points: before intervention (T1) and at 3 subsequent intervals of approximately 4–5 months each (T2, T3, and T4, respectively). For girls who received one component (HC or RC), interventions were conducted between T1 and T2. Girls in RC + HC received RC between T1 and T2 and HC between T2 and T3. In the control condition (SC), girls attended school as they usually would, attending no groups, and completing assessments during the same time points as girls in the intervention conditions.

**Fig. 2:**
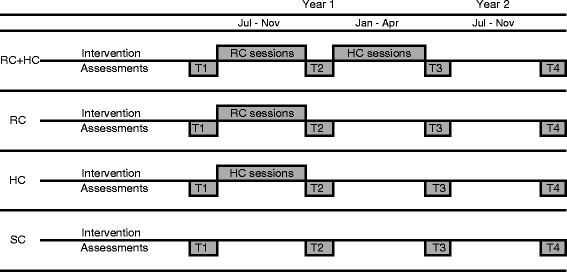
Schedule of interventions and assessments. Legend: HC, Girls First Health Curriculum; RC, Girls First Resilience Curriculum; SC, school-as-usual control; T1, Time 1; T2, Time 2; T3, Time 3; T4, Time 4

The study’s aim was to measure the effects of Girls First and its components across four life domains: emotional, social, physical, and educational. Additionally, the study was designed investigate both (a) *how much* Girls First and its components affected these life domains, as well as (b) *how or why* Girls First affected these life domains. Therefore, a mixed-methods model was chosen, incorporating quantitative and qualitative assessments at each time point. The same quantitative package was administered throughout; the qualitative measurements differed slightly (see sections below on qualitative methods). Figure 2 details assessment and intervention timing.

### Main quantitative outcomes and measures

Because of logistical constraints, self-report measures were used to assess girls’ emotional, social, and physical outcomes. For educational outcomes, although initial plans included gathering data on attendance and grades through school records in addition to self-report, it was quickly discovered that school records were often inaccurate, missing, or incomplete. Therefore, only self-report measures for educational outcomes were completed.

### Choice and development of quantitative measures

The process of choosing measures began with a literature review to identify measures of the constructs of interest that had been used in similar settings. For education and physical health, a number of surveys and questionnaires had been developed for use among Indian adolescents (for example, the Indian Adolescent Health Questionnaire [41], and the SNEHA Adolescents Gaining Ground evaluation [42]). Based on surveys and questionnaires such as these, questions were selected verbatim and/or adapted based on the cultural setting, reading level, and length appropriateness for this particular population and study. These were translated and back-translated, then underwent 2 pilots among a total of 74 girls from a neighboring area (not included in any other portion of the study).

**Fig. 3:**
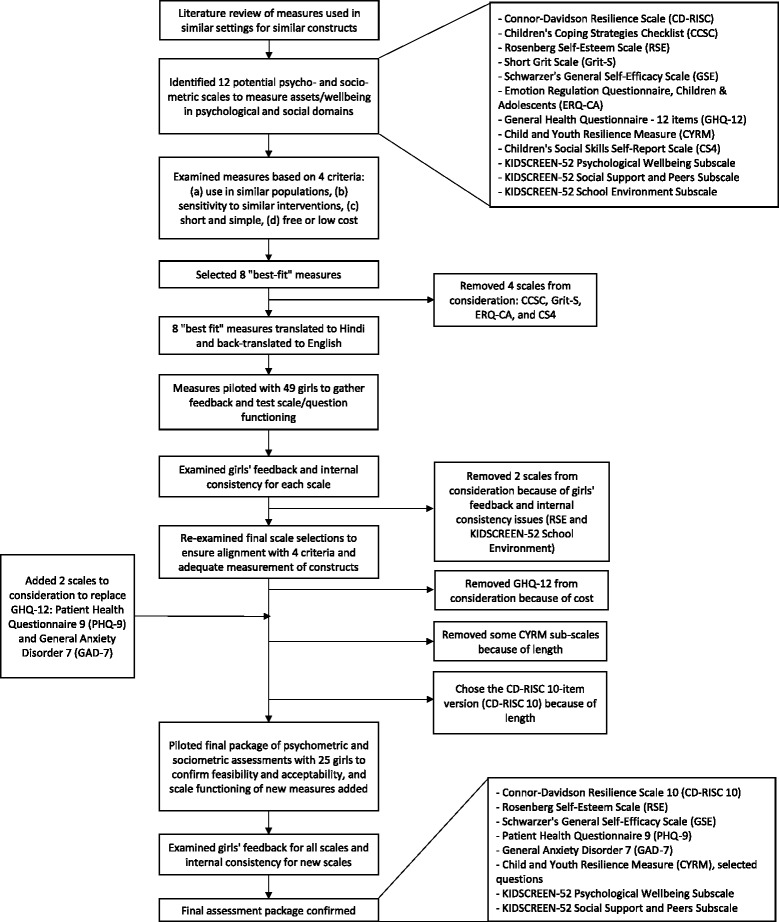
Measurement selection process for emotional and social outcomes

For emotional and social outcomes, the literature review revealed that few assessments had been conducted with high-poverty populations in developing countries. Therefore, the measurement selection and development process included a more in-depth process of measurement selection, translation and back-translation, and piloting. Figure 3 details this process for the emotional and social measures.

### Final quantitative outcome measures

The following outcomes were measured: emotional outcomes (emotional resilience, self-efficacy, positive psychological wellbeing, and psychological distress), social outcomes (social-emotional assets and social wellbeing), physical outcomes (physical health knowledge, health-related behaviors, gender attitudes, and physical wellbeing), and educational outcomes (school attendance and performance). The main quantitative measures contained in the final measurement package are described in detail in Appendix B (see Additional file 1).

### Quantitative data collection

Before T1, facilitators were trained to administer quantitative assessments, then administered them at T1 with groups of girls with whom they would later conduct group sessions. At T2–4, facilitators conducted assessments with groups of girls they had not facilitated previously, and were not explicitly told girls’ condition assignments in order to reduce bias. This precaution was not deemed necessary at T1 as facilitators did not have any relationships with girls and did not have any experience conducting the intervention.

### Qualitative component procedure

The qualitative component of this study comprised individual interviews and focus group discussions with girls and facilitators in order to contextualize the quantitative data that were gathered. Interviews and focus groups were semi-structured and prioritized open-ended questions.

### Qualitative component aims and frameworks

Throughout the qualitative portion of the study, the aim was to elicit and document: (a) rich descriptions of girls’ experiences in the interventions, (b) narratives of girls’ lives before, during and after participation, (c) girls’ own interpretations and descriptions of how the interventions had affected them, and (d) a “how” in addition to the “how much” that the quantitative component provides (e.g., answering the qualitative question: “*How* are girls becoming more resilient?” in addition to the quantitative: “*How much* are girls becoming more resilient?”).

These goals and the overall approach to the qualitative component drew from multiple frameworks, including ethnography and phenomenology. For instance, drawing from ethnography, it was considered central to understand the unique socio-cultural location of participants as inseparable from intervention effects; notice and document connections among outcome domains; and examine how girls’ actions make sense to them based on their socio-cultural contexts. Drawing from phenomenological techniques [43], rich descriptions and narratives of girls’ subjective lived experiences were privileged – and the meanings that the girls personally attributed to these experiences – as important indications of how the interventions were functioning on a personal level.

### Qualitative component sampling

Forty-six individual interviews and 13 focus groups were conducted with a subset of 99 girls and 27 facilitators over the course of the study. Interviews and focus groups were approximately 1 hour each and were conducted for girls and facilitators separately.

Based on the qualitative component goals and approaches, it was planned that girl participants would be selected to participate in the qualitative portion through mixed purposive and critical case sampling. Given that a major goal of the qualitative component was to document in-depth, experienced mechanisms of change, participants were purposively sampled who were able to describe their experiences and thoughts in an in-depth fashion. Additionally, critical case sampling was used, in which girls who had overcome particularly difficult challenges over the course of the study (e.g., preventing child marriages) were targeted, which helped to examine how resilience was functioning.

Girl interviews and focus groups were conducted at the same four time points as quantitative assessments. Some girls were followed for multiple time points while others participated in only one discussion or interview. For additional information about sample size and other girl interview/discussion considerations, please see Appendix C (in Additional file 1).

Facilitators were included as an exploratory component of the study. They were reached only at T2 and T4. As the major goal in including facilitators in the study was for them to discuss their experiences and observations of the girls, accessing them at T1 would not have been useful as they were not involved with the girls at that time. Resource constraints limited access to facilitators to one other time point only; therefore, T4 was chosen as facilitators would be able to reflect on their experiences both at T3 and T4. Table 2 includes details on the number of girls and facilitators accessed at each time point.

**Table 2:** Number of interviews and focus group discussions

Time point	Number of interviews		Number of focus groups	
	Girls	Facilitators	Girls	Facilitators
T1	12	0	5	0
T2	10	5	2	3
T3	7	0	3	0
T4	8	4	0	0

### Qualitative component outcomes and measures

Girl interviews and focus groups included questions mirroring outcomes from the quantitative assessments, eliciting more complete descriptions of girls’ experiences and views related to their background, social life, physical health, emotions and attitudes, and education. Appendix D includes sample questions (in Additional file 1).

At T2–4, additional questions were asked about how the girl felt the program had affected her life, how well sessions connected to her life, and how her responses to any of the topics discussed during the interview might have changed over the course of her program participation. Similar topics and questions were discussed during focus groups with girls, though with less emphasis on personal stories and more emphasis on group experiences and opinions.

The exploratory interviews and focus groups with facilitators served a dual purpose: first, to explore how facilitators noticed or understood the program’s effects among participants, questions were asked about changes they noticed in the girls. Second, to explore the effects of facilitating the program on facilitators’ lives, similar questions to those used among girls were asked of facilitators.

### Qualitative data collection

Interviews and focus groups were conducted in Hindi by Master Trainers or other trained staff members at GENVP and IDF. Interviews and focus groups were recorded, transcribed, and translated by bilingual Hindi-English translators.

## Results and discussion

This intervention trial tested a school-based intervention with potential to contribute to the evidence surrounding improving emotional, social, physical and educational wellbeing among adolescent girls in developing countries such as India.

This trial randomized a set of 76 schools, 19 in each of 4 arms. Additionally, a set of 74 local women with a Std. X education were trained and monitored to facilitate the program. Quantitative data were collected from 3,560 girls at 4 time points with very high rates of participant retention (91–93 % of the sample was reached at each time point). Qualitative assessments were conducted with a subset of 99 girls and 27 facilitators.

There are four guiding principles that facilitated trial implementation: (a) a commitment to integrating diverse sources of knowledge and expertise throughout, (b) flexibility and speed of planning, monitoring, and implementation, (c) prioritizing measurement selection and development, and (d) finding a balance between rigor and feasibility.

### Integrating diverse sources of knowledge

This study was mixed-methods and multi-disciplinary in many senses, as it integrated not only quantitative and qualitative methods, but also methods from multiple fields and disciplines in designing the intervention and measures, and significant expertise and input from both local and non-local sources. These two major means of integrating diverse knowledge are detailed below.

### Drawing from multiple disciplines for a holistic trial

This trial drew from fields as diverse as restorative practices, international adolescent health, and emotional competence in developing the intervention. In developing quantitative measures, learnings and expertise from adolescent health, psychology, and education were also integrated. This commitment to using multiple sources of expertise and knowledge was critical in developing a truly holistic intervention and set of measures: for instance, targeting multiple life domains (psychological, social, educational, and physical) during one intervention trial necessitates the ability to translate among relevant bodies of work and previous knowledge.

### Prioritizing local knowledge for cultural relevance

Local staff and others familiar with the population were also integral throughout the trial in providing feedback on interventions, questionnaires and making decisions about what to measure from a standpoint of ensuring cultural appropriateness. The addition of qualitative measures was also important in order to respect and include local and girl-level explanations and understandings of change alongside quantitative results. Local staff were also integral in developing training, support, and monitoring strategies that were culturally relevant and appropriate. Using local knowledge alongside non-local knowledge was important in not only ensuring feasibility, accessibility, and high quality of interventions and measures, but also in ensuring co-ownership of study procedures and results.

### Flexibility and speed of planning, monitoring, and implementation

There were many challenges throughout the trial that required flexible and fast responses, many of which were discovered through close monitoring, documentation and reporting. For example, some facilitators found trainings more difficult to grasp than others and required more refresher trainings than had originally been planned. However, through monitoring protocols, these issues were quickly discovered and refresher training schedules were quickly adjusted in order to maintain high intervention quality.

Another example occurred during sampling. Initially, official enrollment records at each school were used to estimate the number of students who would be present the following year. However, this turned out to be a poor approximation, as many fewer students actually enrolled than were present in official records. Again, this was discovered through close monitoring and process documentation. In consultation with local staff, it was determined that in order to achieve the sample size desired, additional schools should be selected. Though this was not originally planned, these schools were added without delay. Without close monitoring as well as the ability to regroup and quickly adjust protocols as appropriate, this adjustment would not have been possible.

### Prioritizing measurement selections

The measurement selection process was very intensive (see Fig. 3). One of the largest barriers for researchers working on emotional and social issues in LMICs is measurement. Even if an intervention affects an outcome, there are many reasons that a previously-used measurement tool may not reflect that impact. For instance, a tool may not be sensitive to the intervention’s effects in that population, may not measure the same construct in that population as in others, or may be linguistically or culturally confusing.

In part using expertise drawn from multiple sources, including advice from local staff and local and non-local experts in adolescent health and psychology measurement, a measurement selection and development protocol was created and followed that aimed to reduce this risk. Providing details about this process is also critical, as others who wish to measure effects on emotional and social outcomes in developing countries can adapt this process for their specific situation and improve upon it.

### Balancing rigor and feasibility

Throughout this trial, rigorous scientific practices were prioritized in order to maintain high data quality and high internal and external validity of results. As many who have worked outside the laboratory know, however, rigor and real-world feasibility do not always work together well: the best, most scientifically rigorous practice may be impossible logistically, financially, culturally, or for some other real-world reason. The usual conflict between rigor and real-world feasibility can be further amplified by the additional challenges of working in a high-poverty or low-income population in an LMIC, such as fewer available human and financial resources, logistical difficulties such as challenging transportation, and cultural differences among populations that could change interpretations of interventions and assessments.

A balance between rigor and real-world feasibility was critical not only for trial implementation but also for imagining how lessons learned could be useful in the future. Specifically, a major study consideration was to follow processes that were not so resource-heavy, logistically-complex or culturally-specific as to be prohibitive in the future for others in similar settings. For example, the choice of school sites was not dictated only by random selection as this was unlikely to mirror future logistical constraints on organizations in similar areas. Instead, local organizations were asked to choose those schools with whom they felt comfortable working to form the study frame. Then, within that set of schools, schools were randomly assigned to conditions. Additionally, the choices of measures included emphases on low-to-no-cost tools with evidence of use in diverse populations, in hopes that the selected measurements could be usable in the future by others in similar populations with similarly constrained budgets and resources.

## Conclusions

The Girls First intervention trial provides a critical example of testing an innovative intervention to improve adolescent girls’ emotional, social, physical, and educational wellbeing in an LMIC. Not only the intervention but also the methods used in evaluating the intervention are important in building the evidence base surrounding empowering girls and women worldwide. The Girls First trial has shown the importance of considerations such as integrating diverse sources of knowledge and expertise; remaining flexible in planning, monitoring, and implementation; being careful about measurement selection; and striking a balance between rigor and feasibility. This trial has the potential to provide a much-needed basis for future studies in this area.

## Additional file

Additional file 1:
**All appendices referenced in this article are contained in this additional file.** (DOCX 48 kb)

## Abbreviations

CD-RISC 10: Connor-Davidson Resilience Scale 10-item
CONSORT: Consolidated Standards of Reporting Trials
CYRM-28: Child and Youth Resilience Measure 28-item
GAD-7: General Anxiety Disorder 7-item
GEM: Gender Equitable Measurement Scale
GENVP: Gramin Evam Nagar Vikas Parishad
HC: Girls First Health Curriculum
HIC: higher income country
ICRW: International Center for Research on Women
IDF: Integrated Development Foundation
LMIC: low and middle income country
PHQ-9: Patient Health Questionnaire 9-item
RC: Girls First Resilience Curriculum
RCT: randomized controlled trial
SC: school-as-usual control group
SD: standard deviation
SDQ: Strengths and Difficulties Questionnaire
Std.: standard (grade level in the Indian school system)
T1: T2, T3, T4, Time 1, Time 2, Time 3, Time 4 (respectively)

## Footnotes

1 Although the standard age for Stds. VII–VIII in India is approximately 12–14 years, in reality girls are often younger or older than their grade suggests. In this sample, although most girls were within the range of 12–14 years of age, girls in these grades were as young as 9 years and as old as 18 years (Mean = 13.0 years, SD = 1.2 years) at Time 1.

2 The minimum was set as 20 girls as this was the minimum number required to form a single intervention group given potential non-response and attendance issues. The maximum of 150 was chosen in order to most closely reflect the rural school environments where Girls First was most likely to be run in the future. Given that the average size of an entire rural school in India is 244 students, [44] a school with more than 150 girls across only 2 grade levels was considered to be well above that average and, therefore, poor proxies for the future rural school environments for Girls First.
