# Supplementary material for: Fostering emotional, social, physical and educational wellbeing in rural India: the methods of a multi-arm randomized controlled trial of Girls First
Source: Trials. 2015 Oct 26;16:481. doi: 10.1186/s13063-015-1008-3 (PMC4620631; doi:10.1186/s13063-015-1008-3)
Supplement: Additional file 1: — All appendices referenced in this article are contained in this additional file. (DOCX 48 kb) [file 13063_2015_1008_MOESM1_ESM.docx]

# Additional File 1 – Appendices

# Appendix A – Intervention Details

Girls First is based on evidence from the following fields:

(1) positive psychology [1]

(2) emotional competence/intelligence [2]

(3) restorative practices [3–6]

(4) global adolescent health [7]

(5) peer support [8–10]

Girls First Resilience Curriculum (RC). The RC integrates components from fields 1-3 above and includes 23 hour-long sessions. This portion of the curriculum aims to increase internal assets (such as self-efficacy, coping skills, health knowledge, and conflict-resolution skills) and external assets (such as positive bonds with peers and adult mentors).

The RC was developed to be culturally flexible; for instance, girls discuss concepts that have been found to be relatively universal across cultures (for instance, their character strengths [11]), and are then encouraged to share examples from their own lives to make the sessions culturally and personally relevant. Additionally, the curriculum was designed to work from the “inside out”; starting by giving girls a sense of their strengths, goals, and emotions, and then moving on to applying that knowledge and their newfound skills to social relationships, conflict resolution, and group problem solving.

During the first RC sessions, girls identify and practice using their character strengths [12] (evidence base 1; positive psychology) then use their knowledge of these strengths to plan to reach their goals. The curriculum then moves on to teach coping skills, drawn from positive psychology methods such as “benefit finding” [13] (evidence base 1), and emotional intelligence methods [2] such as identifying and managing difficult emotions (evidence base 2).

The RC then provides girls with a space to use the strengths and skills developed during the first portion of the curriculum to solve concrete problems in their lives and resolve conflicts (this portion of the curriculum draws largely on restorative practices; evidence base 3) [14]. The final project – the “peace project” – asks girls to integrate and exercise what they have learned throughout the RC to create a project to increase peace in their own or others’ lives in a way that is meaningful to them (following the understanding, developed most recently through positive psychology, that meaning is an important aspect of wellbeing [15]).

Girls First Health Curriculum (HC). The HC provides adolescent physical health education, drawing from international adolescent health intervention strategies (evidence base 4). This 21-session curriculum provided in-depth training in physical health and wellness topics such as sexual and reproductive health, common diseases, nutrition, gender equality, and substance use. These were previously- and locally-identified priority areas for adolescent health in Bihar, identified through interviews and program reviews with local stakeholders and potential beneficiaries that CorStone conducted in 2012. The HC was adapted in large part from an adolescent health training called Adolescents Gaining Ground that has been successfully conducted among adolescents in Mumbai by an organization called SNEHA [16].

Social and educational development. Throughout both components, social wellbeing development is targeted with the peer support format of the groups (evidence base 5), which aims to strengthen girls’ social environments.

Additionally, although neither component (RC or HC) directly targets educational outcomes, both components have a theoretical relationship with improved education, as in previous studies, physical health and social and emotional wellbeing have been linked to educational outcomes [17, 18].

# Appendix B – Quantitative Measure Details

## Emotional Outcomes

### Emotional resilience.

Emotional resilience includes multiple assets that allow a girl to bounce back from and overcome challenges, including hardiness, persistence, focus under pressure, flexibility and adaptability in the face of change, and ability to handle difficult emotions. Emotional resilience was measured using the 10-item version of the Connor-Davidson Resilience Scale (CD-RISC 10) [19]. In the measurement pilot sample, this scale had Cronbach’s α = 0.91.

### Self-efficacy.

Self-efficacy is the belief in one’s ability to achieve goals and influence the outcome of events in one’s life [20–22]. Self-efficacy is an important asset, shown in previous studies to be closely related to resilience. For instance, it predicts proactive behaviors in overcoming challenges as daunting as homelessness [23], and even predicts the occupational levels children reach as adults [24]. Self-efficacy was measured using Schwarzer’s General Self-Efficacy Scale [25]. This scale had internal consistency of α = 0.90 in the pilot.

### Positive psychological wellbeing.

Positive aspects of psychological wellbeing, including positive emotions and life satisfaction, were measured using the Psychological Wellbeing subscale from the KIDSCREEN-52 Quality of Life Measure for Children and Adolescents [26]. This subscale has evidence of cross-cultural use with generally very good psychometric properties; however, this evidence is mostly from European countries [26] with some successful use of this particular subscale among children and adolescents in Latin America [27] and East Asia [28]. In the pilot sample, this scale had α = 0.92.

### Psychological distress.

Psychological distress was defined as a combination of symptoms common in mental health problems such as depression and anxiety, which was measured with a combination of the Patient Health Questionnaire-9 (PHQ-9) [29] and the General Anxiety Disorder-7 (GAD-7) [30]. These scales have been used in many populations worldwide as well as among adolescents with excellent psychometrics [29–33]. In the pilot sample, the scales had α = 0.78 (depression) and α = 0.92 (anxiety).

## Social Outcomes

### Social-emotional assets.

A number of items from the Child and Youth Resilience Measure 28-item version (CYRM-28) were selected that measured social-emotional assets [34]. Item choices were made in consultation with staff at GENVP and IDF about which assets were most important for girls in this particular culture, as well as which assets were most likely to be influenced by the RC. These included social assets such as cooperation, knowledge of how to behave in different social situations, and desire to help out in the community; as well as assets stemming from relationships in girls’ lives, such as being treated fairly by others and having positive role models. Questions 1-4, 12, 16, 19-21, 23, and 25 were included from the original CYRM-28. In the pilot sample, the selected items had α = 0.92.

### Social wellbeing.

Girls’ peer relationships were examined as a critical indicator of social wellbeing because of the peer support component of the interventions. Social wellbeing among peers was measured using the Social Support and Peers subscale of the KIDSCREEN-52 [26]. This subscale has been widely used with excellent psychometric properties, especially in Europe among children and adolescents, though there have also been some tests in non-European countries as well [26, 28]. In the pilot sample, this scale had α = 0.81.

## Physical Outcomes

### Physical health knowledge.

Physical health knowledge was assessed through a 14-point multiple choice test of knowledge taught during HC, including topics like pregnancy, HIV, nutrition, malaria, and legal issues for women. This test was developed specifically for the study based on the knowledge that girls would gain through the HC. Girls received one point for each correct answer; thus, the range of possible scores was 0-14. Sample items include: “You can prevent anemia by: A – Chewing on ice, B – Drinking black tea, C – Eating iron-rich food, D – All of the above, E – I don’t know” and “Which of the following could be negative consequences of a girl getting married before age 18?: A – Discontinuing her education, B – Being at a greater risk of facing violence from her husband, C – Increased risk of death during childbirth, D – All of the above, E – I don’t know.” Higher scores indicate greater knowledge.

### Health-related behaviors.

A number of health-related behaviors were measured, including both risky and beneficial behaviors. Questions covered practices related to clean water, menstrual hygiene, doctor visits, nutrition, etc. Some items were adapted from previously-used evaluations (including the Indian Adolescent Health Questionnaire [35] and SNEHA Mumbai’s Adolescents Gaining Ground evaluation [16]). Others were developed specifically for this study. Sample questions included: “During the past month, how often were you able to wash your hands before eating?” (from the Indian Adolescent Health Questionnaire) and “When you get your period, how many times do you usually change the cloth/sanitary napkin/tampon each day?” (developed for this study). All questions were multiple choice.

### Gender attitudes.

Gender equality attitudes were considered part of the physical health outcomes measured because they are traditionally targeted by many other international adolescent health programs [e.g., 16]. The current adolescent health curriculum (HC) included attention to gender equality and gender roles, as well as important legal issues surrounding gender relationships (i.e., the legality of a husband beating his wife, what the legal age of marriage is for boys vs. girls, etc.). RC did not include any emphasis on these facts, thus the construct’s placement as a “physical health” outcome.

Attitudes related to gender equality were measured using a gender attitudes scale that was developed specifically for this particular cultural setting and age group, loosely based on the Gender Equitable Measurement (GEM) Scale from the Gender Equity Movement in Schools of the International Center for Research on Women (ICRW) [36]. The scale consisted of eight statements about gender equality on which girls indicated their agreement, using a 5-point Likert scale from 0 (strongly agree) to 4 (strongly disagree). Sample items included: “There are times when a woman deserves to be beaten,” and “Educating girls is as important as educating boys.” The range of possible scores was 0-32, with higher scores indicating greater equality in gender attitudes.

### Physical wellbeing.

Physical wellbeing was measured through a number of different variables: vitality, energy, safety, substance use, etc. Some questions were drawn from previously-used questionnaires, including the KIDSCREEN-52 [26], the Indian Adolescent Health Questionnaire [35], and the SNEHA Adolescents Gaining Ground evaluation [16]. Others were developed or significantly adapted for this study. Sample questions include: “Do you feel safe when at home?” (from the Indian Adolescent Health Questionnaire) and “In the last week, have you felt fit and well?” (from the KIDSCREEN-52). All questions were multiple choice.

## Educational Outcomes

### School attendance.

Girls were asked to think about the last month that they were in school and report how many days they had missed for various reasons, such as days missed home or family obligations, problems with students or teachers, or menstruation.

### School performance.

School performance was measured with the self-report question: “If you are currently studying, how do you think you are doing in school compared to others in your class?” rated on a scale of 1 (very well) to 5 (very poorly).

# Appendix C – Girl Interview and Focus Group Discussion Considerations

In determining sample size, the goal was to gather about eight detailed cases through interviews for girls (about two per arm) at each time point, and to hold four focus groups to elicit a general understanding of girls’ lives in the area (about one per arm) at each time point.

We originally planned to conduct 7-8 interviews and four focus groups at each time point, distributed across arms, to reach this goal. However, while in the field a number of adjustments were made. First, more girl interviews were conducted at T1 because the level of detail in the interviews was lower than expected (potentially because girls had not participated in any intervention yet at T1 which would help them feel comfortable opening up, or because staff did not know the girls well enough yet to appropriately select eloquent participants).

Second, girl focus groups were emphasized less and less throughout the study. It was found that girls were often not very descriptive and tended to simply agree with one another when they were in a group, while they were much more confident in expressing their own opinions and perceptions in an interview setting. Therefore, only 10 focus groups were conducted rather than the 12 anticipated.

Finally, fewer SC girls were included than originally planned (four interviews rather than eight over the course of the study) as the data that emerged was quite similar throughout. Girls in SC generally did not express major changes in their lives or describe very different issues over the course of the study. Their interviews were quite similar to those conducted among girls at T1, as these girls had not received any intervention at that time, either. It was therefore decided to include fewer SC girls than originally planned and to use that time to include more intervention girls.

# Appendix D – Sample Qualitative Interview and Focus Group Questions

Interview and focus group discussion guides included questions about:

- participants’ backgrounds and daily lives (e.g., “Can you describe what you do on a typical day?”)
- attitudes and experiences with education (e.g., “What do you enjoy about school?”)
- difficulties and problem solving (e.g., “Can you tell me the story of a time that was very difficult for you, and how did you get through that time?”)
- aspirations and hopes (e.g., “Think about what you want your life to be like 10 years from now, and describe it in as much detail as you can.”)
- social lives (e.g., “In your family, neighborhood and school, who do you get along with the best and why?”)
- physical health (e.g., “Have you ever had to go to the doctor or take medicine for a health problem? What did you call the problem and how did it happen?”)

# References

1. Seligman MEP, Ernst RM, Gillham J, Reivich K, Linkins M: **Positive education: positive psychology and classroom interventions**. *Oxf Rev Educ* 2009, **35**:293–311.

2. Goleman D: *Emotional Intelligence*. 10th Anniversary Hardcover. New York, NY: Bantam Books; 2006.

3. IIRP: **Improving School Climate: Findings from Schools Implementing Restorative Practices**. 2009.

4. Sumner M, Silverman C, Frampton ML: **School-based Restorative Justice as an Alternative to Zero-tolerance policies: Lessons from West Oakland**. 2010.

5. Freudenberg N, Ruglis J: **Reframing school dropout as a public health issue**. *Prev Chronic Dis* 2007, **4**.

6. Ruglis J, Freudenberg N: **Toward a Healthy High Schools Movement: Strategies for Mobilizing Public Health for Educational Reform**. *Am J Public Health* 2010, **100**:1565–1570.

7. Kirby DB, Laris BA, Rolleri LA: **Sex and HIV Education Programs: Their Impact on Sexual Behaviors of Young People Throughout the World**. *J Adolesc Health* 2007, **40**:206–217.

8. Benard B: **Fostering Resiliency in Kids: Protective Factors in the Family, School, and Community.** 1991.

9. Ellis LA, Marsh HW, Craven RG: **Addressing the challenges faced by early adolescents: a mixed-method evaluation of the benefits of peer support**. *Am J Community Psychol* 2009, **44**:54–75.

10. Cowen EL: **The enhancement of psychological wellness: challenges and opportunities**. *Am J Community Psychol* 1994, **22**:149–179.

11. Park N, Peterson C: **Moral competence and character strengths among adolescents: The development and validation of the Values in Action Inventory of Strengths for Youth**. *J Adolesc* 2006, **29**:891–909. [*Special Issue: New Methodological Directions for the Study of Adolescent Competence and Adaptation*]

12. Peterson C, Seligman MEP: *Character Strengths and Virtues: A Handbook and Classification*. 1 edition. Washington, DC : New York: American Psychological Association / Oxford University Press; 2004.

13. Tennen H, Affleck G: **Benefit-finding and benefit-reminding.** In *Handbook of Positive Psychology*. Edited by Snyder CR, Lopez SJ. New York, NY: Oxford University Press; 2002:584–597.

14. McCluskey G, Lloyd G, Kane J, Riddell S, Stead J, Weedon E: **Can restorative practices in schools make a difference?**. *Educ Rev* 2008, **60**:405–417.

15. Seligman MEP: *Flourish: A Visionary New Understanding of Happiness and Well-Being*. Reprint edition. New York: Atria Books; 2012.

16. SNEHA Mumbai: *Adolescent Health and Empowerment at SNEHA: Adolescents Gaining Ground and Arogyamitra Kendra*. Mumbai, India: SNEHA; 2013:1–43.

17. Suldo S, Thalji A, Ferron J: **Longitudinal academic outcomes predicted by early adolescents’ subjective well-being, psychopathology, and mental health status yielded from a dual factor model**. *J Posit Psychol* 2011, **6**:17–30.

18. Trockel MT, Barnes MD, Egget DL: **Health-Related Variables and Academic Performance Among First-Year College Students: Implications for Sleep and Other Behaviors**. *J Am Coll Health* 2000, **49**:125.

19. Campbell-Sills L, Stein MB: **Psychometric analysis and refinement of the connor–davidson resilience scale (CD-RISC): Validation of a 10-item measure of resilience**. *J Trauma Stress* 2007, **20**:1019–1028.

20. Bandura A: **Self-efficacy: Toward a unifying theory of behavioral change**. *Psychol Rev* 1977, **84**:191–215.

21. Bandura A: *Social Foundations of Thought and Action: A Social Cognitive Theory*. 1 edition. Englewood Cliffs, N.J: Prentice Hall; 1985.

22. Bandura A: *Self-Efficacy: The Exercise of Control*. Macmillan; 1997.

23. Epel ES, Bandura A, Zimbardo PG: **Escaping Homelessness: The Influences of Self-Efficacy and Time Perspective on Coping With Homelessness1**. *J Appl Soc Psychol* 1999, **29**:575–596.

24. Bandura A, Barbaranelli C, Caprara GV, Pastorelli C: **Self-efficacy beliefs as shapers of children’s aspirations and career trajectories**. *Child Dev* 2001, **72**:187–206.

25. Schwarzer R, Jerusalem M: **Generalized Self-Efficacy scale**. In *Measures in health psychology: A user’s portfolio. Causal and control beliefs*. Windsor, England: NFER-NELSON; 1995:35–37.

26. Ravens-Sieberer U, Gosch A, Rajmil L, Erhart M, Bruil J, Power M, Duer W, Auquier P, Cloetta B, Czemy L, Mazur J, Czimbalmos A, Tountas Y, Hagquist C, Kilroe J: **The KIDSCREEN-52 Quality of Life Measure for Children and Adolescents: Psychometric Results from a Cross-Cultural Survey in 13 European Countries**. *Value Health* 2008, **11**:645–658.

27. Quiceno JM, Vinaccia S: **Calidad de vida, fortalezas personales, depresión y estrés en adolescentes según sexo y estrato**. *Int J Psychol Psychol Ther* 2014, **14**:155–170.

28. Hong SD, Yang JW, Jang WS, Byun H, Lee MS, Kim HS, Oh M-Y, Kim J-H: **The KIDSCREEN-52 quality of life measure for children and adolescents (KIDSCREEN-52-HRQOL): reliability and validity of the Korean version**. *J Korean Med Sci* 2007, **22**:446–452.

29. Kroenke K, Spitzer RL, Williams JB: **The PHQ-9: validity of a brief depression severity measure**. *J Gen Intern Med* 2001, **16**:606–613.

30. Spitzer RL, Kroenke K, Williams JBW, Löwe B: **A brief measure for assessing generalized anxiety disorder: the GAD-7**. *Arch Intern Med* 2006, **166**:1092–1097.

31. Löwe B, Decker O, Müller S, Brähler E, Schellberg D, Herzog W, Herzberg PY: **Validation and Standardization of the Generalized Anxiety Disorder Screener (GAD-7) in the General Population**. *Med Care* 2008, **46**:266–274.

32. Lotrakul M, Sumrithe S, Saipanish R: **Reliability and validity of the Thai version of the PHQ-9**. *BMC Psychiatry* 2008, **8**:46.

33. Yu X, Tam WWS, Wong PTK, Lam TH, Stewart SM: **The Patient Health Questionnaire-9 for measuring depressive symptoms among the general population in Hong Kong**. *Compr Psychiatry* 2012, **53**:95–102.

34. Resilience Research Centre: *The Child and Youth Resilience Measure-28*. Halifax, NS: Dalhousie University; 2008.

35. Long KNG, Long PM, Pinto S, Crookston BT, Gren LH, Mihalopoulos NL, Dickerson TT, Alder SC: **Development and validation of the Indian Adolescent Health Questionnaire**. *J Trop Pediatr* 2013, **59**:231–242.

36. Achyut P, Bhatla N, Khandekar S, Maitra S, Verma RK: *Building Support for Gender Equality Among Young Adolescents in School: Findings from Mumbai, India*. New Delhi: International Center for Research on Women (ICRW); 2011:1–12.
